# Preliminary study of hypoxia-related cardiovascular mediator-markers in patients with end-stage renal disease with and without diabetes and the effects of haemodialysis

**DOI:** 10.1371/journal.pone.0178171

**Published:** 2017-05-23

**Authors:** A. Treweeke, J. Hall, S. Lambie, S. J. Leslie, I. L. Megson, S. M. MacRury

**Affiliations:** 1 Division of Health Research, University of the Highlands and Islands, Inverness, Scotland; 2 Department of Medicine, Raigmore Hospital, Inverness, Scotland; Hospital Universitario de la Princesa, SPAIN

## Abstract

**Background:**

Evidence points to activation of pro-inflammatory and pro-thrombotic stimuli during the haemodialysis process in end-stage renal disease (ESRD) with potential to predispose to cardiovascular events. Diabetes is associated with a higher incidence of cardiovascular disease in haemodialysis patients. We tested the hypothesis that a range of mediators and markers that modulate cardiovascular risk are elevated in haemodialysis patients with diabetes compared to those without.

**Methods:**

Men and women with diabetes (n = 6) and without diabetes (n = 6) aged 18–90 years receiving haemodialysis were recruited. Blood samples were collected and analysed pre- and post-haemodialysis sessions for (platelet-monocyte conjugates (PMC), oxidised LDL (Ox-LDL), endothelin 1 (ET-1) and vascular endothelial growth factor (VEGF-A).

**Results:**

PMC levels significantly increased after haemodialysis in both groups (diabetes p = 0.047; non-diabetes p = 0.005). Baseline VEGF-A was significantly higher in people with diabetes (p = 0.009) and post-dialysis levels were significantly reduced in both groups (P = 0.002). Ox-LDL and CRP concentrations were not significantly different between groups nor affected in either group post-dialysis. Similarly, ET-1 concentrations were comparable in all patients at baseline, with no change post-dialysis in either group.

**Conclusions:**

In this pilot study, we have confirmed that circulating PMCs are increased following dialysis irrespective of diabetes status. This is likely to be a mechanistic process and offers a potential explanation for high rates of vascular events associated with haemodialysis. The higher VEGF-A concentrations between patients with and without diabetes is a previously unreported finding in diabetic ESRD. Further research is merited to establish whether VEGF-A is a marker or mediator (or both) of cardiovascular risk in haemodialysis.

## Introduction

Diabetes associated end-stage renal disease is the most common cause of new patients requiring renal replacement therapy in developed countries [1,2], with a continuing rise in cases documented in the United States and an adjusted prevalence rate currently estimated at > 750 per million [3]. Patients with diabetes have a high incidence of macrovascular disease, including cardiovascular, cerebrovascular and peripheral arterial disease (PAD) [4]. The risk of cardiovascular disease is amplified in chronic kidney disease (CKD) [5] and, in patients with both ESRD and diabetes, the prevalence of ischaemic heart disease and congestive cardiac failure are increased by 78% and 100% respectively over ESRD patients without diabetes [6]. Haemodialysis is the most frequently used form of renal replacement therapy in patients with end-stage renal disease (ESRD) but is also recognised to increase the risk of cardiovascular events [7]. The elevated risk is disproportionately higher in patients with diabetes [8], but the reasons for this increased risk are not fully understood. Dialysis treatment has been independently identified to be associated with foot ulceration in patients with diabetes [9], although the causes of this remain obscure. Relative hypoxia induced by lowering of blood pressure, coupled with macro- and micro-vascular dysfunction could be one explanation predisposing to vascular events. Hypoxia can induce platelet activation [10], which is likely to predispose to increased platelet-monocyte interaction, a marker of cardiovascular event risk [11–14]. Furthermore, heparin used routinely in dialysis can have paradoxical pro-thrombotic effects through induction of hypercoagulability associated with heparin-induced thrombocytopaenia, and it has been hypothesised that this may also be related to depression of endothelium-derived tissue factor pathway inhibitor (TFPI) [15,16]. Inflammatory cell activation may result in increased levels of inflammatory cytokines, whilst hypoxia induces a number of adverse reactions, including endothelial dysfunction [17,18], inflammatory cytokine release and increased levels of reactive oxygen species (ROS), which in turn induce platelet activation [19].

Given the high profile role for hypoxia in the manifestations of both diabetes and PAD, the aim of this pilot study was to test the hypothesis that mediator markers of hypoxia (vascular endothelial growth factor; VEGF-A), endothelial function (endothelin-1; ET-1) and oxidative stress (oxidised low density lipoprotein; ox-LDL), are higher in haemodialysis patients with diabetes compared to those without diabetes.

## Materials and methods

### Study participants

Men and women (n = 12) aged >18 years receiving haemodialysis were recruited from the renal unit at Raigmore Hospital in Inverness between August 2013 and March 2014. Study visits coincided with routine dialysis sessions. They comprised six people with diabetes (type 1 (n = 4) or type 2 (n = 2)) and six people without diabetes (absence of diabetes defined as HbA1c level <48mmol/mol (6.5%)). Duration of diabetes ranged from 8–42 years. All participants had been receiving haemodialysis therapy for >6 months to ensure a stable pattern of dialysis had been established. Current smokers or those stopped for <6 months were excluded.

All volunteers provided written informed consent. The North of Scotland Research ethics service approved the study (13/NS/0005) which complied with the Declaration of Helsinki and its amendments.

### Dialysis

All patients were receiving dialysis three times weekly. Adequacy of dialysis was assessed by urea reduction ratio (URR) and Kt/V (dialyser clearance of urea x time/ total body volume). The duration of dialysis session (start to finish) was also recorded. Body Mass Index (BMI) Wt (kg)/ Ht (m^2^) as calculated from post dialysis weight.

Blood pressure was estimated pre- and post-dialysis on the dialysis machine.

### Blood samples

Blood was collected in EDTA tubes pre dialysis and analysed for haemoglobin, haematocrit and ferritin levels as measures of anaemia status and for glycated haemoglobin as a measure of glycaemic control using a liquid chromatography methods (HPLC, Tosoh Bioscience) on Diabetes Control and Complications Trial (DCCT) aligned equipment.

Additional blood samples were collected into heparin tubes pre- and post-dialysis which were either centrifuged at 5000*g* to prepare plasma for storage at -80^°^C, or the whole blood used for platelet-monocyte conjugate (PMC) measurement.

### Flow cytometry

PMC measurements by flow cytometry were performed as previously described [20]. Briefly, 50μl of heparinised whole blood was incubated with CD14-FITC and CD41-PE-Cy5 antibodies, or the appropriate isotype controls for 15 min. After addition of 0.5ml FACS lysing solution (Becton Dickinson (BD), Oxford, UK), the cells were incubated for a further 15 min and then immediately analysed for conjugates using a FACSCalibur flow cytometer (BD). Dual-stained PMCs were identified as CD14/CD41–positive events expressed as a percentage of total CD14 positive cells.

### Laboratory assays

Frozen plasma was thawed and used to measure Ox-LDL, ET-1, VEGF-A and creatinine using commercial kits. Each assay was performed according to the manufacturer’s instructions. Ox-LDL was used as a marker of oxidative stress, using a competitive ELISA kit, (Mercodia, Diagenics, Milton Keynes, UK) [21]. ET-1, assayed with a solid phase Quantikine ELISA kit (R&D systems, Abingdon, UK) and VEGF-A, measured using a Platinum ELISA, (eBioscience, Hatfield, UK) were used as markers of endothelial dysfunction. Plasma CRP levels were measured using the Quantikine ELISA Human C reactive protein/CRP immunoassay kit (R&D systems), according to the manufacturer’s instructions.

Samples were corrected for serum creatinine level using a colorimetric assay, (Cayman Medical, Cambridge Bioscience, UK).

### Statistical analysis

Use of parametric vs nonparametric statistics was determined according to whether groups had equal variances and were normally distributed (Kolmorov-Smirnov test; version 5.00, Graphpad, San Diego, CA). Student’s t-test and Willcoxon matched pairs test were used for normally distributed and non-normally distributed data respectively. Patient characteristics between groups and presented as median ± IQR and are compared using Mann-Whitney U test. Two-factor data were compared using a two-factor ANOVA. P<0.050 was accepted as statistically significant.

## Results

### Baseline participant characteristics

There were no baseline differences in body mass index or the anaemia indicators (haemoglobin, haematocrit) between the diabetes and non-diabetes groups, although serum ferritin level was higher in the diabetes group. Length of dialysis sessions varied between 2.5 to 4.0 hours and there were no differences between the URR and Kt/V values between the groups (Table 1). Median HbA1c in the diabetes group was 51 (IQR: 42–72) mmol/mol and 33 (normal range < 48) mmol/mol in the non-diabetes group.

**Table 1:** Baseline characteristics of the groups.

	Diabetes(n = 6)	No Diabetes(n = 6)	P value
Age(yr)	54.5(51.0–78.2)	82.5(75.5–84.2)	0.030
Body Mass Index(kg/m^2^)	23.9(22.9–26.9)	22.8(21.9–24.2)	0.310
Haemoglobin(g/L)	115(111–122)	109(106–116)	0.260
Haematocrit	0.360(0.325–0.378)	0.334(0.323–0.354)	0.470
Ferriitn(ng/ml)	421(341–533)	260(202–318)	0.021
Duration of dialysis(yr)	3.75(3.25–4.0)	3.75 (3.5–4.0)	0.850
Urea reduction ratio(URR)	73.5(67.0–80.0)	75.0(72.7–76.0)	0.740
Dialyser clearance ratio(Kt/V)	1.32(1.20–1.50)	1.36(1.21–1.47)	0.810

All participants (with and without diabetes) had evidence of cardiovascular disease as documented in individual patient case records, including ischaemic heart disease, cerebrovascular disease or PAD. Four patients were receiving aspirin therapy alone (2 with and 2 without diabetes), two receiving clopridogrel alone (both diabetes), three were receiving warfarin (1 with and 2 without diabetes), one patient with diabetes was receiving both aspirin and warfarin and three patients were not receiving any anti-platelet or anticoagulant therapy (1 with and 2 without diabetes).

Four of the patients were receiving blood pressure reducing therapy (2 with diabetes of which one patient was receiving β-Blocker therapy alone and one receiving both a calcium channel blocking agent with an angiotensin II receptor antagonist and 2 without diabetes: one receiving a combination of β-blocker and an angiotensin-converting enzyme inhibitor therapies and the other receiving a calcium channel blocking agent along with an angiotensin-converting enzyme Inhibitor). There were no significant changes in systolic or diastolic BP between or within groups before or after dialysis.

### Platelet-monocyte conjugates

**Fig 1:**
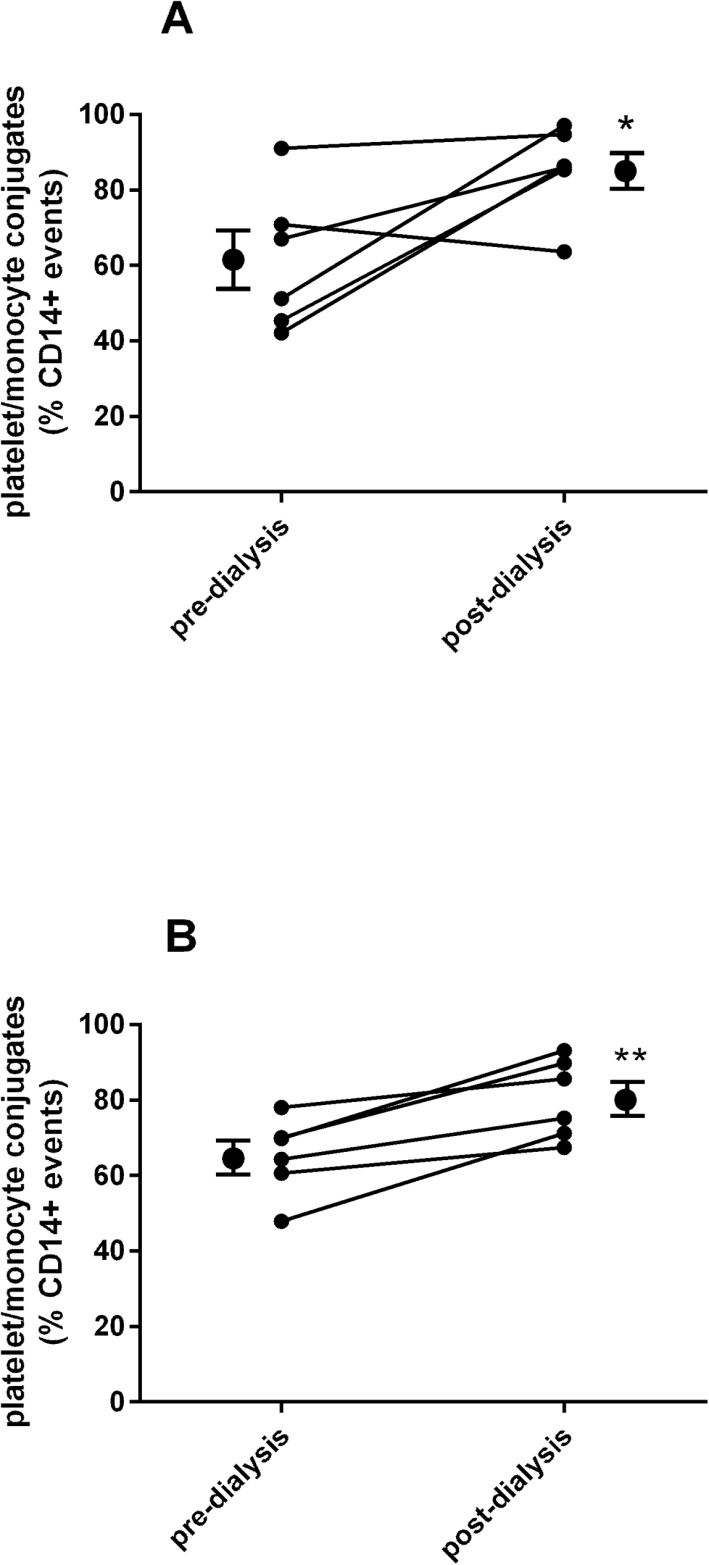
Platelet-monocyte conjugates pre and post dialysis in patients with (A) and without diabetes (B).

PMC levels were higher at baseline and post dialysis in all participants relative to published values for type 2 diabetes patients [15,16]. Following dialysis, PMC levels were significantly increased in both groups (diabetes, P = 0.047; non-diabetes, P = 0.005; Fig 1).

### Hypoxia-related markers

**Fig 2:**
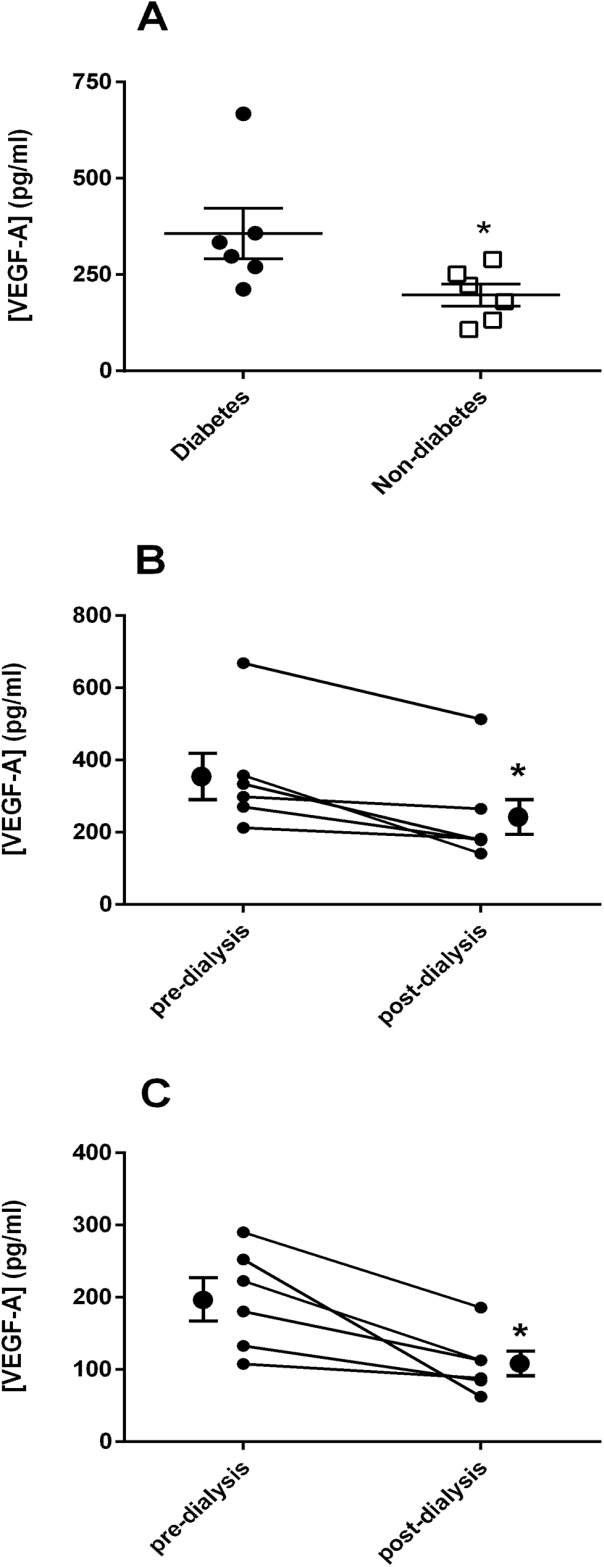
VEGF-A levels at baseline (A), and pre- and post-dialysis in diabetes (B) and non-diabetes (C).

**Fig 3:**
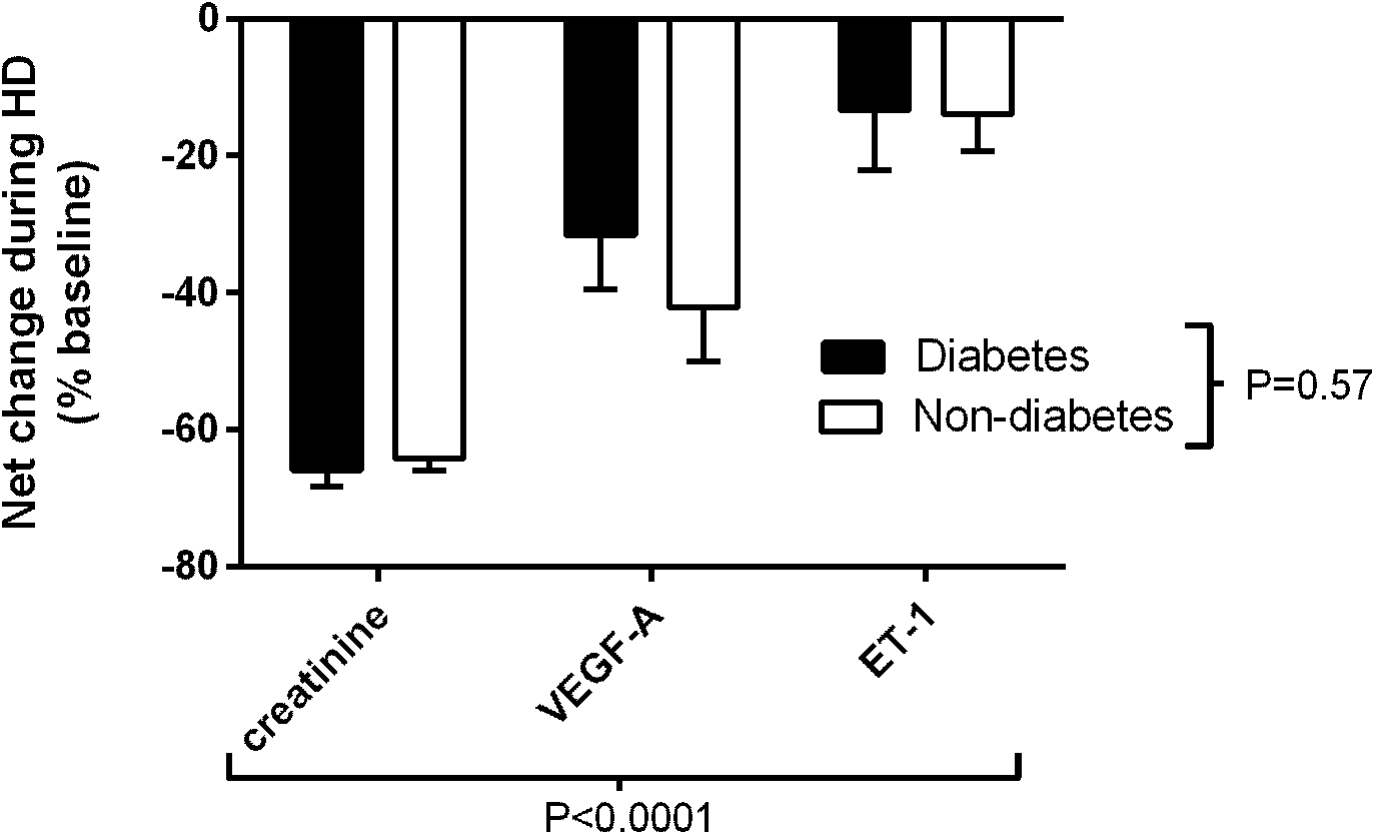
Net changes in creatinine, VEGF-A and Endothelin-1 (ET-1) during haemodialysis in patients with and without diabetes (2-way ANOVA).

Baseline VEGF-A levels were significantly higher in people with diabetes (P = 0.026; Fig 2) and were significantly reduced post dialysis in both groups (diabetes, P = 0.047; non-diabetes, P = 0.031; Fig 2), with no difference between the groups. Ox-LDL and CRP levels were similar pre-haemodialysis (p = 0.630 Ox-LDL and p = 0.267 CRP) and unchanged for both groups post dialysis. Similarly, there was no difference in ET-1 levels between the groups pre or post-dialysis, Fig 3.

## Discussion

The aim of this pilot study was to measure some markers of cardiovascular risk and function immediately prior to and after haemodialysis in ESRD patients with and without diabetes, with a view to exploring the potential influence of specific pathways on the increased risk of cardiovascular events in patients with diabetes. The results confirmed that the haemodialysis procedure increased prevalence of PMC markers of cardiovascular risk, but that there was no difference in these markers between patients with and without diabetes. Of the markers selected to establish the potential role of various pathological pathways in increased risk in haemodialysis patients, ox-LDL and ET-1 were not found to be different between patients with and without diabetes, but VEGF-A was significantly elevated in diabetes patients prior to haemodialysis.

PMC is gaining recognition as an effective marker for cardiovascular risk [11–14]]. Elevated PMC is indicative of an increased level of circulating platelet activation that might predispose to thrombus. Moreover, conjugation of activated platelets to monocytes acts to propagate activation of the monocytes themselves, increasing the potential for monocyte interaction with the endothelium [14] an early critical event in the atherogenic process [22].

Patients with ESRD have an increased cardiovascular risk profile which is likely to be multi-factorial [23]. However, while haemodialysis appears to have an impact on inflammation and oxidative stress [24], the exact cause is unknown.

Our results demonstrate that PMC levels are raised in patients with ESRD and are augmented following the dialysis process, perhaps indicative of the established cardiovascular disease of these individuals. The extent of PMCs was striking in these ESRD patients; even the baseline PMC counts were found to be higher than reported levels in people with type 2 diabetes, a group known to have a high prevalence of vascular complications [11]. Indeed, they were similar to those for patients with MI [14]. However, the PMC counts were not significantly different between the diabetes and non-diabetes groups, suggesting that diabetes status is not a determinant of the extent of PMC formation. The lack of difference between the groups might, however, reflect the higher median age amongst the non-diabetes group, although evidence is lacking to support this. Equally, differences in anti-platelet drug therapy between the groups is a possible confounder, with the diabetes group generally receiving more anti-platelet therapy than the non-diabetes group. PMC have been assessed previously with regard to patients with ESRD receiving haemodialysis therapy and are raised, probably as a result of multiple interacting cellular factors. Although dependency on the type of dialyser membrane has been suggested as another possible determinant of PMCs, [25] this is less likely with the dialyser membrane in use in our unit, which is made from helixone, a synthetic polysulfone. The rising levels post dialysis in this study may reflect a number of factors related to the dialysis process, including hypoxia, hypotension or perhaps an adverse effect of the dialysis tubing or membrane on platelet or monocyte activation. It would appear that a mechanical effect is responsible for the change in level at the time of dialysis. Patients had been receiving dialysis for a minimum of six months and the baseline levels could reflect a step change; it would be worth measuring PMC across the spectrum of renal impairment to the pre-dialysis stage to ascertain if there is an incremental rise that inversely correlates with degree of renal impairment and if renal transplantation has a positive benefit. Nevertheless, our results offer a possible explanation for the higher rate of vascular problems associated with dialysis in patients with diabetes.

VEGF-A is a mediator of angiogenesis on vascular endothelial cells and correlates with measures of endothelial damage and dysfunction [26]. Glycated haemoglobin (HbA1c) is an independent predictor of VEGF concentration and a reduction in both HbA1c and LDL cholesterol levels are associated with a reduction in VEGF levels in people with diabetes with and without cardiovascular disease [27]. VEGF appears to be protective in kidney disease, where it maintains structure through tissue repair and fibrogenesis [28], although it is also linked to the development of diabetic nephropathy [29]. Interestingly, studies suggest that VEGF-A concentration tends to fall with progression of diabetic nephropathy [30]. In incident haemodialysis in patients with ESRD of all aetiologies, a longitudinal study found no difference in levels of VEGF compared with those not receiving dialysis or indeed health control subjects when starting dialysis or after 12 months. However, higher levels of VEGF were associated with an increase in all-cause mortality [31]. While a number of cytokines, chemokines, growth factors and physico-chemical conditions such as hypoxia and oxidative stress regulate VEGF-A, the higher level in ESRD in people with diabetes in this study compared to those without is unexplained. Raised blood glucose through creation of a hyperglycaemic mediated pseudo-hypoxic state may induce VEGF-A production [27]. Although our patients appeared to have reasonable glycaemic control, glycated haemoglobin is an unreliable marker of glycaemia in diabetic ESRD. The lower levels of VEGF-A post-dialysis in common with the fall in ET-1 levels probably reflect dialysis-mediated clearance. VEGF-A is a mediator of microvascular disease in diabetes but it remains unclear whether VEGF-A is a marker or mediator (or both) of diabetic ESRD and cardiovascular complications. Further studies are necessary to validate our findings and to elucidate the role of VEGF-A in ESRD.

Oxidised LDL is a validated marker of oxidative stress. However, while we were unable to demonstrate any significant changes in Ox-LDL, possibly due the small sample size, this does not preclude the possibility that ROS is increased during the dialysis process and that other markers may reflect this [32]. The role of Nitric Oxide in particular represents a future area of interest with respect to haemodialysis and cardiovascular outcomes.

Antioxidant effects are associated with some antihypertensive therapies nonetheless, it is difficult to determine individual antioxidant activity given the relationship between increased oxidative stress and response to blood pressure lowering [33,34]. Given that blood pressure was well controlled in the participants, that few were receiving blood pressure lowering therapy and that these were equal across the groups, it is unlikely that antihypertensive therapy had any impact on the markers measured.

Endothelin-1, derived mainly from endothelial cells, is a potent vasoconstrictor and marker of endothelial dysfunction [33]. Increased production mediates the development of atherosclerosis and vascular complications in diabetes [35] and levels have been shown to be raised post dialysis in hypertensive patients, perhaps reflecting increased systemic vascular resistance related to endothelial dysfunction [36]. Hypertension was not a significant feature associated with the patients in this study and the lower levels of ET-1 obtained post-dialysis are likely to reflect dialysis-mediated clearance.

### Limitations of the study

This was a small, hypothesis generating study. Nevertheless, the study was sufficiently robust to confirm high circulating PMC levels in patients with ESRD, irrespective of diabetes and also to suggest that VEGF-A is significantly higher in ESRD patients with diabetes pre-dialysis. We acknowledge, however, that the study could be underpowered to identify more subtle changes in the other markers that we measured (ET-1 and ox-LDL). Ideally, we would have preferred to have age-matched the groups, but this proved impossible on account of the demographic of the ESRD patients with and without diabetes undergoing dialysis. Perhaps it is unsurprising that the average age of patients with diabetes undergoing dialysis is substantially lower than those without–the UK renal registry indicates that age of people with diabetes starting renal replacement therapy in Scotland is younger than those without and with reduced survival probably accounting for some of the age difference [1].

## Conclusion

In this study we have confirmed that PMC are increased in patients with ESRD receiving dialysis therapy, but not shown any difference in this marker between patients with and without diabetes. Further, dialysis appears to increase PMC, probably via a mechanical process. This may represent one explanation to account for the high rate of vascular events experienced by patients on dialysis. The significant elevation of VEGF-A in patients with diabetes and ESRD is a novel finding. At this stage, it is unclear whether VEGF-A is simply acting as a marker or whether it might contribute to elevated risk of cardiovascular events in patients with the diabetes and ESRD.
